# Health Equity Impact Assessment (HEIA) reporting tool: developing a checklist for policymakers

**DOI:** 10.1186/s12939-023-02031-0

**Published:** 2023-11-18

**Authors:** Alireza Olyaeemanesh, Amirhossein Takian, Hakimeh Mostafavi, Mohammadreza Mobinizadeh, Ahad Bakhtiari, Fateme Yaftian, Abbass Vosoogh-Moghaddam, Efat Mohamadi

**Affiliations:** 1 Health Equity Research Center (HERC), TehranUniversity of Medical Sciences (TUMS), Tehran, Iran; 2 National Institute for Health Research, TehranUniversity of Medical Sciences (TUMS), Tehran, Iran; 3 Department of Global Health and Public Policy, School of Public Health, Tehran University of Medical Sciences (TUMS), Tehran, Iran; 4 Department of Health Management, Policy, and Economics, School of Public Health, Tehran University of Medical Sciences (TUMS), Tehran, Iran; 5 Governance and Health Training and Research Department, National Institute for Health Research, TehranUniversity of Medical Sciences (TUMS), Tehran, Iran; 6 Secretariat for Health and Food Security, TehranUniversity of Medical Sciences (TUMS), Tehran, Iran

**Keywords:** Health Equity, Inequality in health, Health Equity Impact Assessment (HEIA), Health Impact Assessment (HIA), Health Policy

## Abstract

**Introduction:**

Health Equity Impact Assessment (HEIA) is a decision support tool that shows users how a new program, policy, or innovation affects health equity in different population groups**.** Various HEIA reporting and dissemination tools are available, nevertheless, a practical standard tool to present the results of HEIA in an appropriate period to policymakers is lacking. This work reports the development of a tool (a checklist) for HEIA reporting at the decision-making level, aiming to promote the application of HEIA evidence for improving health equity.

**Methods:**

This is a mixed-method study that was carried out over four stages in 2022–2023: 1) identifying HEIA models, checklists, and reporting instruments; 2) development of the initial HEIA reporting checklist; 3) checklist validation; and 4) piloting the checklist. We also analyzed the Face, CVR, and CVI validity of the tool.

**Results:**

We developed the initial checklist through analysis of 53 included studies and the opinions of experts. The final checklist comprised five sections: policy introduction (eight subsections), managing the HEIA of policy (seven subsections), scope of the affected population (three subsections), HEIA results (seven subsections), and recommendations (three subsections).

**Conclusion:**

Needs assessment, monitoring during implementation, health impact assessment, and other tools such as monitoring outcome reports, appraisals, and checklists are all methods for assessing health equity impact. Other equity-focused indicators, such as the equity lens and equity appraisal, may have slightly different goals than the HEIA. Similarly, the formats for presenting and publishing HEIA reports might vary, depending on the target population and the importance of the report.

**Supplementary Information:**

The online version contains supplementary material available at 10.1186/s12939-023-02031-0.

## Introduction

Following the strategies proposed by the World Health Organization (WHO) to improve population health in the member states, i.e. primary health care, health for all (1978), and strengthening health systems (2000) [1], since 2012, universal health coverage (UHC) has been emphasized as the fundamental strategy to consider health for all and enhance health equity [2]. Health inequalities among various populations and societies continue to be a significant challenge for health systems at the national and international levels, despite improvements and developments in health indicators across countries [3–6]. Most of these inequalities interact complexly with disparities in Social Determinants of Health (SDH), i.e., biological, behavioral, environmental, and socioeconomic variables [7–9].

Living in unequal social circumstances results from substantial contextual factors, e.g., inappropriate social programs and policies, disadvantageous economic arrangements, and poor governance [10–12]. The significant gap between the privileged people and the majority of global citizens, who have kept behind in fulfilling their optimal wellness, has occurred because of combination of policies, economics, and politics [13] that have failed to account for SDH, hence health equity is compromised [7]. Health equity and including Health in All Policies (HiAP) are the two requirements for improving SDH [14, 15]. Health is the pillar for sustainable societies, hence the essential requirement to place health on the agenda of all public sectors and make them all accountable for the effects of their decisions on people's health [16]. Health impact assessment (HIA) tools can help minimize any adverse effects on health and maximize the positive effects before policies and programs are implemented [17]. As a subcategory of HIA, HEIA assesses health impacts with a health equity lens. HEIA is a decision support tool that shows the users how a new program, policy, or innovation affects different population groups' health [18]. Equity-focused Health Impact Assessment (EFHIA) uses HIA to develop a complementary and structured method for assessing the potential derivative and distributional impact of a policy or action on the health of a population or a specific group of that population. It also assesses whether the derivative effects are unequal or not. Performing HEIA helps decision-makers identify unanticipated and systematic inequities that may exist in policies and practices. This framework is created for those who are in the position of reviewing an existing or potential policy or action and can alter or influence it [19].

In recent years, several experiences of many countries that developed HEIA have been published [20]. However, there has been less research focused on the assessment reporting method and they weren’t able to provide researchers and policy-makers with a comprehensive tool.

Nevertheless, providing standard public tools and checklists that can briefly present the results of HEIA for policymakers has not been prioritized. Making public policymakers aware of the impact of policies implementation on health inequalities is not only a research priority, but also it is a crucial executive mandate. If the tool is designed in line with the policymakers’ value system and requirements, the results can significantly influence their attitudes, awareness, and ultimately better decision-making. Such a tool guarantees that decision-makers have access to the crucial data and information they need to make well-informed policy decisions. This research aims to develop an evidence-based tool for assessing and reporting HEIA to decision-makers in different sectors. This work reports the process and product of designing a tool (a checklist) for HEIA reporting at the policy-making level, aiming to promote the application of HEIA evidence for improving health equity.

## Methods

**Fig. 1:**
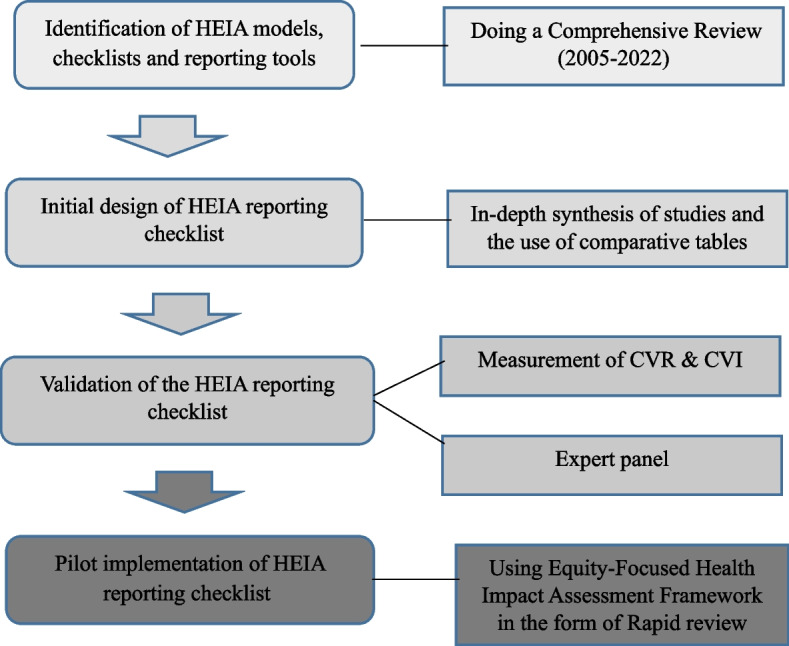
The algorithm for designing the HEIA reporting checklist

The mixed-method study was conducted in the following four stages during 2022–2023: 1) identification of HEIA models, checklists, and reporting tools; 2) initial HEIA reporting checklist design; 3) validation of the checklist; and 4) its pilot implementation (Fig. 1).

### Identification of HEIA models, checklists, and reporting tools

We conducted a comprehensive review to identify available HEIA models, checklists, and reporting tools, as well as related tools. We searched Scopus; PubMed/Medline; and Google Scholar search engines for studies published in English between 2005 and June 30, 2022.

#### Search strategies

The search strategy was defined and implemented separately for each of the investigated databases, It’s Boolean description is as follows:

- ((“Health equity”) OR (“Health inequality”)) AND ((Assessment) OR (Measurement) OR (Evaluation)) AND ((Framework) OR (Tool) OR (toolkit) OR (Checklist)) AND ((Impact) OR (Effect)) AND (Reporting Checklist) AND (“Health system”).

The search strategies were implemented in such a way that each of the above-mentioned terms were included in the titles and/or abstracts of the searched studies.

We also reviewed the reports, frameworks, and studies that were not published in the form of articles by checking the references of the articles included in the study and referring to related websites, such as the WHO website and its regional offices, the Ministry of Health websites of Australia, Canada, and the United States and the website of Centre for Health Equity of Melbourne School of Population and Global Health.

#### Inclusion criteria

Through the screening process, articles with the following characteristics were included:

- Studies that dealt with health equity impact assessment and health impact assessment were included. Studies that provided conceptual tools and models were also included in the analysis. We also included studies that explained health equity, because they were likely to have discussed the dimensions and components of the tools and frameworks.
- Studies at the national, district, or regional scales or those with limited samples were included regardless of their design (longitudinal, cross-sectional, cohort, etc.).
- Considering the concept of equity, we included studies that used different aspects and methods of health equity impact assessment in their data analysis.
- Studies that were in the form of protocols or had introduced tools entered the final phase of the analysis.

#### Exclusion criteria

Through the screening process, articles with the following characteristics were excluded:

- In languages other than English.
- Merely addressing the importance of HEIA.
- Literature reviews.
- Assessed and measured health equity indicators.
- Provided frameworks for assessing equity indicators or introducing health equity indicators (we were interested in a framework to examine the impact of politics on health equity).
- Investigated the impact of policies related to health equity on health equity indicators.
- General expression and assessment output, without providing details of implementation steps.
- Addressing HIA obstacles and challenges.
- Provided insufficient information to be used in our research; and
- Provided solutions to promote health equity.

#### Data analysis

Using the inductive narrative analysis method to organize the overall experience of the included study and present the procedure of analyzing data to conclude. We examined the contents of the included studies and reviewed all that met the requirements for inclusion in the final analysis. To examine the background of a situation, narrative analysis focuses on actual narrative stories. Additionally, narrative analysis encourages in-depth case-level examination while keeping the phenomenon's contextual components. The capture of the case-level experience within the contextual circumstance is encouraged by narrative analysis. This kind of analysis enables cases to draw meaning from their own lived experiences [21]. First, we created the article introduction table to enter information about each article. The information on the studies included the Title, Study design, Country, Study Level, Year of publication and Approach, as well as the detailed data of the studies, which was utilized as the major feed of the initial design. It included codes and parts about the steps of implementing the tool, evaluation criteria, the method of implementing the tool, and the stakeholders involved in the tool's implementation. We then analyzed the content of each article based on the study's findings, extracted, and recorded following the objectives of the study and the criteria for inclusion. For doing this, coding narrative blocks was done; the findings in each HEIA article were organized using inductive coding. After that the blokes were grouped by process-event; we organized findings blocks into relevant, comparable cycle phases. The items examined in each study are shown in Table 1. Finally, two members of the research team (EM, HM) extracted a report of the information from the included studies and compiled them in the final phase, to ensure the validity of screening and analyzing the content of studies.

**Table 1:** Items and information examined in the studies

**Title**	**Study design**	**Country**	**Study Level**	**Year of publication**	**Approach**	**Summary results**			
						**Processes and procedures of implementing the tool**	**Examined indicators and criteria**	**The method of implementing the tool**	**HEIA actors**

### Initial design of HEIA reporting checklist

We used the output of the first step for the initial design of the HEIA reporting checklist. By conducting an in-depth synthesis of the studies and using comparative tables (Table 1), we extracted and kept track of the data and content of the included studies, i.e., the requirements and dimensions of comprehensive HEIA reporting. As we said in the previous step, the processes and procedures of implementing the tools, examined indicators and criteria, the method of implementing the tools and HEIA actors were extracted from studies. At this step, we used codes to separate the tale into manageable sections for analysis. Following that, we completed the subsections and their order for the initial design. We took relevant information from each study, which was used to design the preliminary model, while taking into account its implementation details and considerations. The draft HEIA reporting checklist was developed after comparing the models and tools enacted from the studies. To increase the coherence and validity of the initial checklist, the data (in-depth synthesis of studies) were analyzed by two authors (AT, EM).

### Validation of the HEIA reporting checklist

We used the experts’ opinions to validate and finalize the HEIA checklist. Seven experts in the fields of health equity and SDH as well as experts working for organizations that make policy, i.e., the parliament and Ministry of Health and Medical Education (MoHME), reviewed the preliminary checklist. Face validity and content validity of the checklist were verified by three dimensions (Table 2); it was created as a questionnaire with five main sections and twenty-five subsections. The content validity ratio and content validity index were calculated by formulas 1 and 2 which are presented in Additional file 1: Appendix 1.

**Table 2:** Dimension of face and content validity and related criteria

Dimension of face and content validity	Criteria
Analyses of face validity for each item through two options	It is Clear and expressive
	Not clear and expressive
Analyses of content validity index (CVI) for each item through five options:	Totally disproportionate
	Disproportionate
	Somewhat proportionate
	Proportionate
	Totally proportionate
Analyses of content validity ratio (CVR) for each item through three options:	Necessary
	Useful but not necessary
	Unnecessary

Two authors (MrM, FY) conducted face-to-face interviews and asked participants to complete a questionnaire about their thoughts on the HEIA reporting checklist. The main sections of the checklist all received scores of at least > 0.79 for the content validity index (CVI). We then determined the content validity ratio (CVR) for each section was determined. Since 50% of the participants voted for the main sections and sub-sections of the checklist as necessary, they were retained, according to the number of participants at this stage (*n* = 7) and based on the Lawshe table [22]. Finally, we added five sections and 25 subsections after the index validity ratio and content validity ratio were matched. Based on the findings of the face validity, we made some changes in the wording and arrangement of the questionnaire items. The table including the participants’ answers is presented in Additional file 1: Appendix 2.

We discussed findings in a series of study team meetings and compared perspectives on how to implement the experts’ recommendations. Finally, the consensus of the experts was used to decide what adjustments should be made to the checklist. An expert panel was then held with the participants of the previous stage (seven people plus the members of the research team) to ensure validity.

### Pilot implementation of the HEIA reporting checklist

We conducted a pilot implementation of the designed tool to identify and address any potential issues and limitations, enhance the validity of the checklist, and practice its implementation. The research team decided to run the HEIA checklist on the policy of removing subsidies from some basic food items in Iran. We used the procedures and methods outlined in the “Equity-Focused Health Impact Assessment Framework” to conduct this assessment [23], as presented in Table 3.

**Table 3:** Materials and methods of HEIA for policy of Removing subsidies from some basic food products

Steps of HEIA	Methods used in this study
Screening	- Qualitative interview - Using algorithm screening (Additional file 1: Appendix 3)
Scoping	- Rapid review - The scope of this HEIA was determined as below: ◦ Doing a Rapid assessment ◦ Management of HEIA” Health Equity Research Center (HERC), Tehran University of Medical Science (TUMS) ◦ Time duration: 5 weeks ◦ Thematic scope: The policy of Removing the subsidies from some basic food products - HEIA subject scope: According to the assessed policy and the studies conducted in this regard, the following health indicators and issues were considered and assessed: ◦ Growth trend of consumer price index (CPI) ◦ Food inflation rate trend ◦ Amount of food consumption ◦ Change the trend of calories consumed by people ◦ Prevalence of malnutrition ◦ Prevalence of underweight and short stature in children under 5 years old - Geographic Area: people of Iran - Duration of potential impacts: assessment of short-term and long-term impacts
Impact Identification	- Comprehensive review - Qualitative interview
Assessment of Impacts	**Using dynamic systems methods** Library review and qualitative interview methods were used to assess the required data. Then, the prospective analysis method of dynamic systems was applied to show the logical relationship between the studied variables and indicators, during which dynamic systems and loops as well as how they were affected and related were determined. The coefficients and intensity of the impacts of the variables on each other were extracted through the review of the studies, and/or the impact of policy implementation on the defined variables was evaluated and predicted for the coming years
Decision-making and Recommendations	- Comprehensive review - Expert panel

## Results

Our initial search identified 16,901 articles on health impact and health equity assessment, environmental impact assessment, social impact assessment, and measurement of health equity indicators. After removing 16,658 duplicate and irrelevant studies through initial screening, 243 articles were included in the abstract review stage. 144 articles were excluded during the abstract review, 99 entered the entire text examination phase, and 53 entered the final phase of analysis [18, 19, 24–71]. According to the descriptive analysis of the studies, the majority of them were qualitatively carried out (*N* = 26), and the studies in this field were mostly planned and carried out at the national level (*N* = 31). Since 2016 up till the present, evaluation studies of the effects of equity on health have received increased attention. Canada and Australia served as leading countries for undertaking studies on various forms of health outcome evaluations and health equity results (Additional file 1: Appendix 4).

The final HEIA reporting checklist has five sections as follows (Table 4):

- Section I: Policy introduction (eight subsections)
- Section II: Managing the HEIA of policy (seven subsections)
- Section III: Scope of affected population (three subsections)
- Section IV: HEIA Results (seven subsections)
- Section V: Recommendations (three subsections).

**Table 4:**
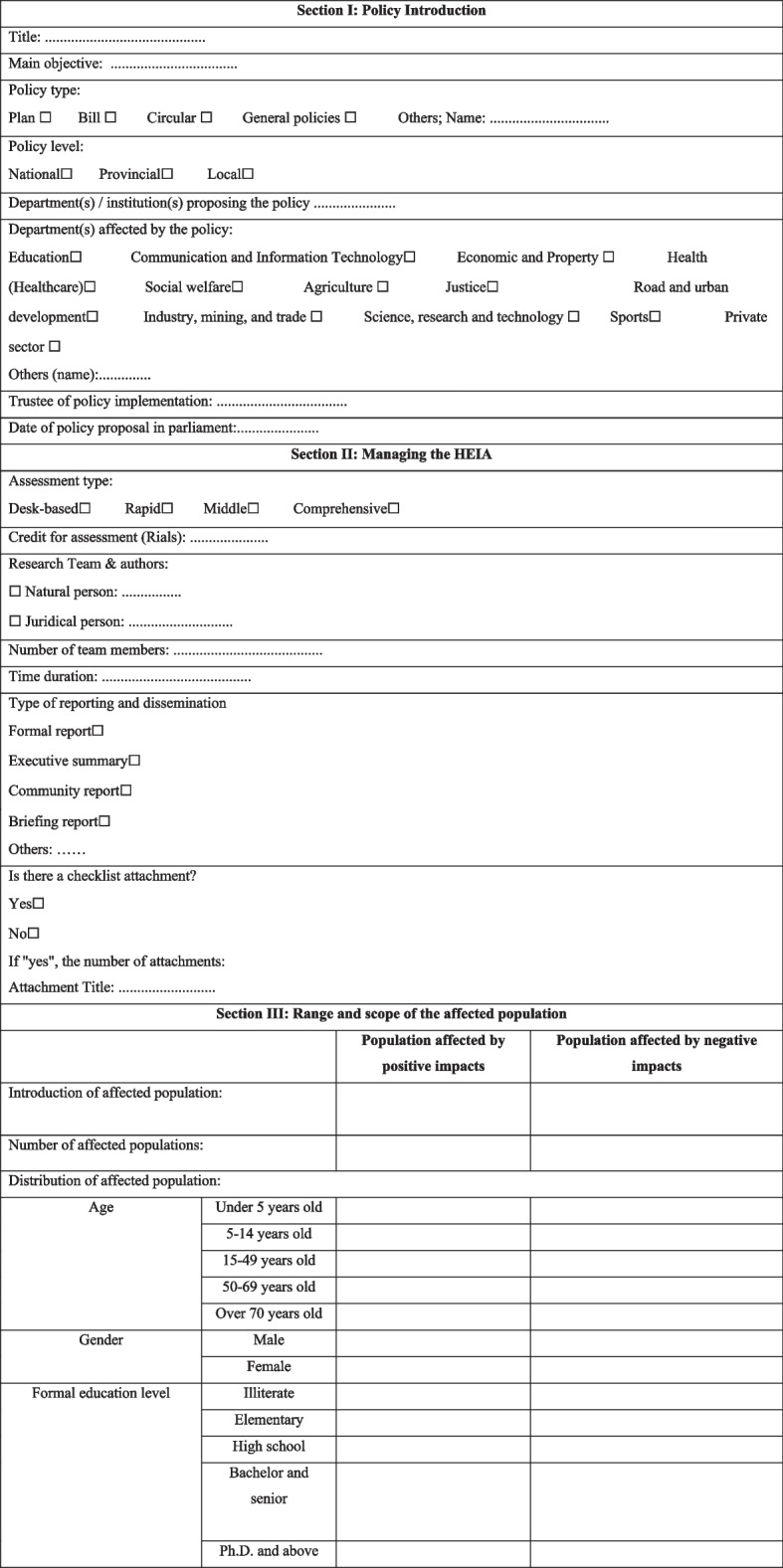

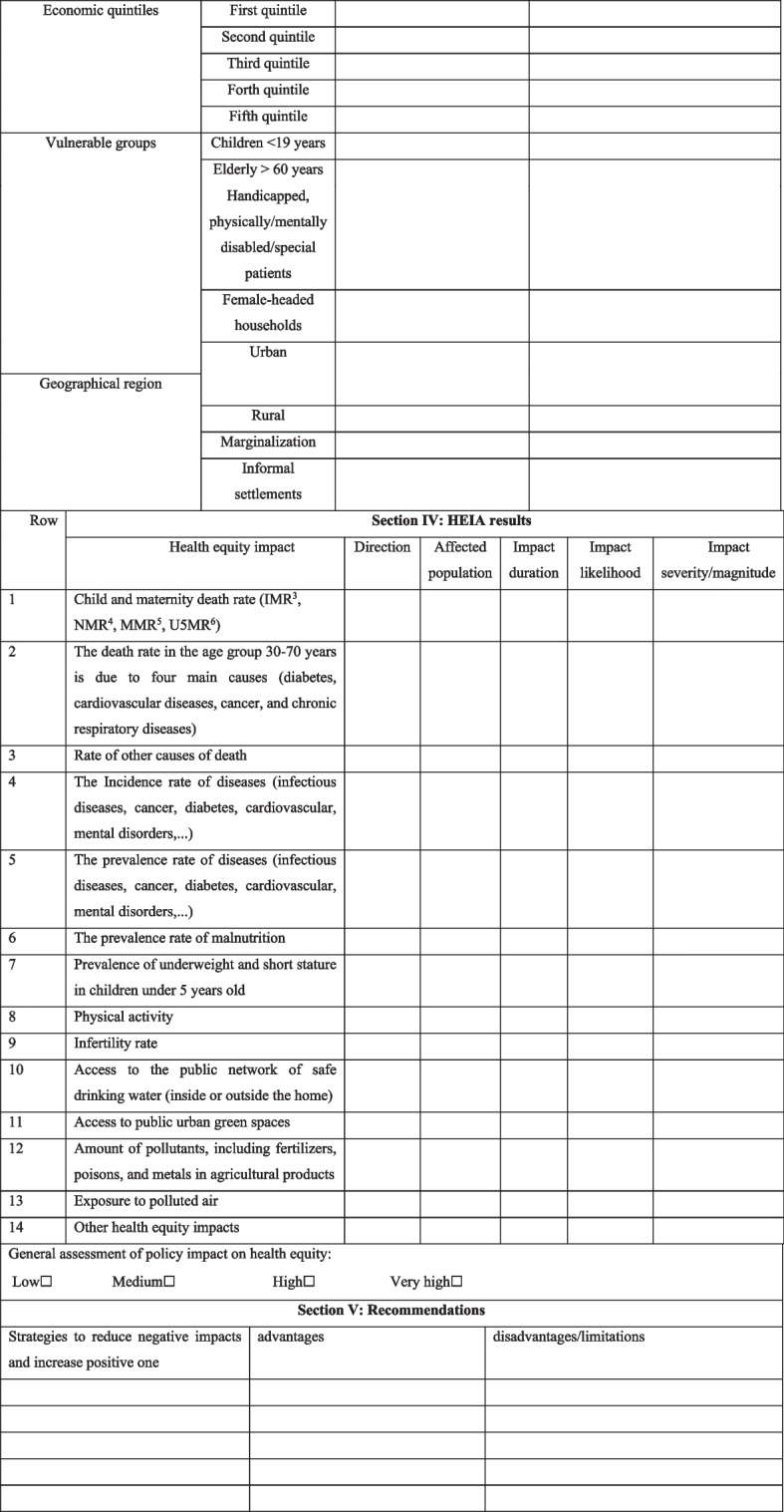
HEIA reporting checklist

^a^Infant Mortality Rate

^b^Neonatal Mortality Rate

^c^Maternal Mortality Rate

^d^Under five Mortality Rate

### Section I

#### Policy introduction

Each checklist was assigned to a policy; which was assessed in terms of its impact on health equity. Therefore, the first section of this checklist provided an introduction to the policy and its various dimensions. The information needed to be added about a policy was as follows:

- Policy: Policy means any document that is supposed to receive the approval of the parliament.
- Title: The exact title of the policy to be approved and is supposed to be evaluated from the perspective of health equity.
- Main goal: The general goal of the policy under review.
- Type of policy: Checklist aimed to ensure that all government approvals considered equity. In this section, the type of proposal and its level were checked.
- Policy level: Determining whether the policy was national, provincial, or local (local meant one or more districts or one or more specific cities (smaller than the province level).
- Department(s) and Institution(s) Proposing a Policy: In this section, the main government department(s) and institution(s) that proposed policy for parliamentary approval were introduced.
- Department(s) Affected by the Policy: Defining the beneficiaries of a policy that might be directly or indirectly affected by its implementation.
- Trustee of policy implementation: The main custodian(s) of implementing the proposed policy.
- Date of policy proposal in the parliament: The time for proposing policy to the parliament.

### Section II

#### Managing the HEIA of policy

The second part of the checklist, entitled “HEIA Management”, provides some information on how the assessment of the policy impact on health equity was carried out.

- Assessment type: Different types of HEIA could be typologically divided into four categories: desk-based HEIA, rapid HEIA, middle HEIA, and comprehensive HEIA (Table 5).
- Credit for conducting the assessment (Iranian Rial): Funding HEIA at different scales, the amount of financial aid, depending on the scope and extent of the assessment.
- Research team & authors: The name and affiliation of the main assessors.
- Number of team members: The number of policy assessment team members involved in field and report compilation.
- Assessment duration: The time spent on HEIA.
- Type of reporting and dissemination: The way that HEIA reports might be presented, i.e., formal reports, executive summaries, community reports, or briefing reports [17].
- Does the checklist have any attachments? Whether the completed checklist has any attachments; as well as the number and title of each attachment.

**Table 5:** HEIA typology based on the scope

desk-based HEIA	rapid HEIA	middle HEIA	comprehensive HEIA
✓ Taking 2 days to 1 week ✓ Providing an overview of potential health impacts	✓ Very fast (1 week to 6 weeks)	✓ 4 weeks to several months	✓ Several months to several years
✓ Based on available documents	✓ Requiring few resources	✓ Requiring a moderate amount of resources	✓ Requiring significant resources
✓ Without beneficiaries’ participation	✓ Without beneficiaries’ participation	✓ Participation of some beneficiaries	✓ Significant participation of beneficiaries
	✓ No need to collect new data	✓ Collecting some new data often relies less on the existing data bank	✓ usually including the collection of primary data

### Section III

#### Scope of affected populations

To determine people affected by the positive and negative consequences of implementing the policy. The information needed to be added about the affected population was as follows:

- Introducing the affected population: The affected population could include ordinary people of a region, a specific group of patients, a specific category of jobs, a specific age group, and/or those with any other characteristics. The section specifies whether the population was affected by the positive or negative impacts of the policy.
- The number of affected populations: The number of affected people for each of the aforementioned groups.
- Distribution of affected population: Distribution of the affected populations by age, sex, education level, economic quintile, vulnerable groups, and geographical region.

### Section IV

#### HEIA results

This section provides a summary of the results of assessing the impacts of selected policies on health equity, which include information about:

- Type: This section deals with the “types” of health equity impacts, referring to the effects of policy implementation on inequalities in health impacts (mortality rates, incidence and prevalence of diseases, etc.).
- Direction: To indicate whether the impact was beneficial or harmful to health equity.
- Affected population: To specify the affected population separately for each of the specified impacts.
- Duration of impact^1^: When or how long does the exposure often occur? The time interval between the implementation of the policy and its effect on each health equity impact was determined and recorded:
  - ✓ One to two years: short-term
  - ✓ Two to five years: medium-term
  - ✓ Over five years: long-term.
- Likelihood of impact: This refers to the likelihood of exposure or impact. Likelihood refers to the strength of the research/evidence, showing causal relationships between policy impacts and health equity impacts: limited evidence, limited but strong evidence, and a causal relationship established. A causal impact meant that the impact was likely to occur regardless of its severity or magnitude.
- Impact severity: This part showed how severe potential health impacts might be:
  - ✓ Low: The effect was not noticeable.
  - ✓ Moderate: The impact results were discomfort, minor injuries, or illnesses that did not require interventions.
  - ✓ High: The impact led to moderate injury or illness that might require interventions.
  - ✓ Very high: The impact led to loss of life, serious injuries, or chronic diseases that required interventions.
- Overall assessment of the impact of policy on health equity: In this part, according to the aforementioned information, the assessor gave his overall judgment about the impact of the policy under review on health equity: low, medium, high, and very high.

### Section V

#### Recommendations

This section provides specific recommendations to modify the conditions for minimizing the anticipated adverse effects of the concerned policy on health equity and maximizing its potential benefits. In the first column of “recommendations”, strategies to reduce negative impacts and increase positive effects are mentioned according to their priority. In the second and third columns, the advantages, disadvantages, and limitations of each solution are mentioned respectively. This might assist policymakers in making the right decisions regarding the implementation of the policy under consideration.

The pilot finding showed that the policy of removing the subsidies from some basic food products has a “high” impact on health equity. Malnutrition prevalence rate, Prevalence of underweight, and Prevalence of short stature are the negative impacts of this policy on health equity which affects most first economic quintiles, vulnerable groups, and children under five years old. Additional file 1: Appendix 5 summarizes the pilot study and the assessment report of “the policy of removing the subsidies from some basic food products” [72–76].

## Discussion

The result was based on numerous evidence from scientific sources. Initially, the most relevant studies to evaluating the impacts of health equity and assessing health outcomes through an equity lens were identified and thoroughly examined. The ultimate objective was to develop a comprehensive tool and checklist for reporting HEIA at the policy-making level. The first draft of the tool was formulated based on the analysis conducted. Subsequent stages involved implementing necessary modifications and ensuring the validation of the tool in collaboration with experts in two phases. In the final phase, the HEIA reporting tool was piloted and refined for practical implementation, with a focus on ensuring its usability and effectiveness.

This tool, which is specifically tailored for transit policy audiences as a “Brief report” [66], incorporates essential requirements and crucial considerations that public policymakers should take into account when formulating policies. It is divided into five components, each of which has a specific function. The first section, named “ Policy introduction”, provides a concise and overarching summary of the policy under evaluation, ensuring that the reader gains a comprehensive understanding of the policy. The second section, titled “Managing the HEIA of policy”, enables the reader to assess various aspects of the evaluation project, such as the involved actors, project implementation approach, and the quality of evidence and results presented. The third and fourth sections (“Scope of affected populations” & “HEIA Results”), which provide in-depth information and analysis of the policy evaluation, make up the tool's main components. In these sections, the tool presents a comprehensive and unique compilation of the policy’s impact on health equity from both positive and negative perspectives. It meticulously analyzes and summarizes the results for different population groups and health equity indicators separately. This approach enables policymakers to consider unforeseen and systemic inequalities that might arise from policies and practices. By providing a comprehensive overview, the tool equips decision-makers with the necessary information to address any potential inequities inherent in the policy. The “Recommendations” as the fifth section of the tool further aids policymakers by offering practical recommendations that are tailored to address specific concerns identified throughout the evaluation process. These recommendations are accompanied by an assessment of their respective strengths and weaknesses. By providing this detailed analysis, the tool assists policymakers in effectively utilizing the suggested solutions during policy revision and decision-making. This ensures that the final decisions are well-informed and take full advantage of the insights provided by the evaluation.

Studies were reviewed, and the results revealed a variety of national experiences [18, 19, 24–71, 77]. One study, “Health equity impact assessment” emphasizes that HIA methods alone cannot provide a comprehensive health equity assessment [18]. Another study titled “Critical considerations for the practical application of health equity tools: providing a conceptual map” highlights that health equity tools are increasing, but less attention has been paid to the fact that these tools are practical and the probability of their implementation and effectiveness [50]. These studies confirm the importance of the tool designed in this article; this sheds light on how to make these tools more practical and applicable in actual settings.

An applied study entitled “Evaluation of Equity-Based Health Impact of the Portuguese Tobacco Control Law” in 2018 in Portugal, was carried out as a rapid assessment. They used quantitative and qualitative methods. The steps implemented in this study were more concise than the steps proposed in our study; They used three stages: screening, evaluation of consequences and presentation and evaluation of proposals [63]. It was also intended to provide a logical model for HEIA, nevertheless, there was no mention of the HEIA results reporting method. Another study conducted in 2019 in America presented five health policy issues to be assessed with the health equity lens. They suggested that a policy assessment model and the level of proposed interventions be designed based on the intended policy level [62]. Likewise, our findings endorse the need for assessments according to the level and importance of the policy. Thus, the financial resources for assessments would be different.

Australia conducted a quick case study of the EFHIA of a health promotion policy implementation program in 2011. The study provided general and specific recommendations on health equity impacts. The EFHIA identified changes in the development and implementation of the program that could potentially occur and provided some solutions. The assessment was conducted using relatively few resources in a short time. Some reported impacts focused on the implementation plan development and an increase in overall attention to health equity. The case study highlighted some factors and preconditions that might maximize the impact of future EFHIAs on decision-making and implementation [69]. Similarly, in the pilot study of the present research, since the assessment was prospective, it was not possible to expand the information analysis and perform a comprehensive assessment. However, it was attempted to provide practical solutions according to the analyses done. "Health Impacts Assessment in the United States of America" is a comprehensive textbook on the concept of HIA and its implementation steps, and provides practical examples. According to this book, the stages of HIA implementation are as follows: screening, scoping, assessment, recommendations, reporting, evaluations, and monitoring [66]. These steps are almost similar to the steps used in our study.

A study entitled “Designing a Toolkit for Assessment of Health in All Policies at a National Scale in Iran” was conducted to identify the indicators related to “health in all policies”. The aim was the systematic development of inter-sectoral cooperation to promote health equity. In total, 14 main and eight contextual factors were extracted to assess equity in all policies [59]. In our study, we attempted to highlight health in all policies by providing a comprehensive tool for HEIA reporting to assess health equity impacts; however, more attention was paid to the dimensions of health inequalities. Another article stated that promoting health equity in health systems was a priority and a challenge worldwide. Health equity tools have been identified as a strategy for integrating health equity considerations into health systems [50].

Studies conducted in this field also sought to provide detailed guidance on how to include health equity in GRADE^2^ in decision-making processes [78]. They advocate special attention to disadvantaged populations when examining health equity and its final effects. Two approaches were proposed to incorporate health equity considerations: 1) assessing the potential impact of interventions on beneficiaries’ rights, and 2) incorporating equity considerations when judging or weighing each piece of evidence with decision-making criteria, with particular attention to consider the impact of recommendations on health equity for remote and underserved settings as well as deprived populations.

The main limitation of our study was the lack of access to certain published studies. We attempted to address this limitation by corresponding with the authors responsible for these articles. During the pilot phase, there was also a limitation in accessing quantitative data, which prevented us from conducting a Comprehensive HEIA. Therefore, we were unable to complete the pilot study beyond a Rapid review. One of the most important factors for the effectiveness of using a checklist is the existence of comprehensive and reliable information systems in countries. With such information systems, conducting these studies can be done at reasonable costs and within a short period of time. Without access to reliable databases, conducting HEIA can become both expensive and time-consuming. This can lead policymakers to perceive it as non-cost-effectiveness, thus limiting the utilization of a checklist in HEIA. Ultimately, this study advocates the use of a checklist for further HEIA and the design of a software platform as a tool for future studies.

## Conclusions

Several methods exist for assessing health equity impact, i.e., assessment/measurement, needs assessment, monitoring during implementation, health outcomes assessment, monitoring outcome reports, appraisals, and checklists. Other equity-focused measures, such as equity lenses and equity appraisal, may have slightly different objectives than the HEIA. Similarly, there are various formats for presenting and publishing HEIA reports, which can vary depending on the target population and the importance of the report. The HEIA reporting method suggested in this study aims to evaluate a specific proposal (either a policy or an activity) within an appropriate stage of its formulation, when there is still a chance to modify it. This checklist could be considered as a tool by health policymakers to advocate Health Equity in All Policies (HEiAP) to increase the positive impacts of interpectoral policies and reduce health inequities.

### Supplementary Information

**Additional file 1:** **Appendix 1.** Calculation of content validity ratio and content validity index. **Appendix 2.** Output of measuring face validity and content validity of HEIA reporting checklist in the first stage of validating the checklist. **Appendix 3.** HEIA screening algorithm. **Appendix 4.** Details of the literature review conducted in the first stage of the study. **Appendix 5.** Results of the pilot study; HEIA report for “the policy of removing the subsidies from some basic food products”.

## Data Availability

Not applicable.

## Abbreviations

CVI: Content Validity Index
CVR: Content Validity Ratio
EFHIA: Equity-focused Health Impact Assessment
GRADE: Grading Recommendations Assessment and Development Evidence
HEIA: Health Equity Impact Assessment
HEiAP: Health Equity in All Policies
HIA: Health Impact Assessment
HiAP: Health in All Policies
MoHME: Ministry of Health and Medical Education
SDH: Social Determinants of Health
UHC: Universal Health Coverage
WHO: World Health Organization

## Footnotes

1 Magnitude/geographic extent.

2 Grading Recommendations Assessment and Development Evidence.
