# Supplementary material for: Health Equity Impact Assessment (HEIA) reporting tool: developing a checklist for policymakers
Source: Int J Equity Health. 2023 Nov 18;22:241. doi: 10.1186/s12939-023-02031-0 (PMC10657117; doi:10.1186/s12939-023-02031-0)
Supplement: Supplementary file 1 — Additional file 1: Appendix 1. Calculation of content validity ratio and content validity index. Appendix 2. Output of measuring face validity and content validity of HEIA reporting checklist in the first stage of validating the checklist. Appendix 3. HEIA screening algorithm. Appendix 4. Details of the literature review conducted in the first stage of the study. Appendix 5. Results of the pilot study; HEIA report for “the policy of removing the subsidies from some basic food products”. [file 12939_2023_2031_MOESM1_ESM.docx]

**Appendixes**

**Appendix 1:** Calculation of content validity ratio and content validity index

- Formula 1: Calculation of content validity ratio

$$CVR:\frac{N Essential-\frac{N}{2}}{\frac{N}{2}}$$

- Formula 2: Calculation of content validity index

$$CVI:\frac{N Relevant+N Totaly Relevant}{N}$$

N: number of experts

**Appendix 2:** Output of measuring face validity and content validity of HEIA reporting checklist in the first stage of validating the checklist

| **Sections of checklist** | **Face validity (clarity)** | | | **Content validity** | | | | | | **CVI** | **CVR** | **Necessity** |
| --- | --- | --- | --- | --- | --- | --- | --- | --- | --- | --- | --- | --- |
|  |  |  |  | **Necessity (CVR)** | | | **Relevance** | | |  |  |  |
|  | Clear | Somewhat clear | Unclear | Necessary | Useful but unnecessary | unnecessary | Relevant | Somewhat relevant | Irrelevant |  |  |  |
| Section I | 2 | 4 | 1 | 7 |  |  | 6 | 1 |  | 100 | 57 | 100 |
| Section II | 6 |  | 1 | 5 | 1 | 1 | 5 | 2 |  | 100 | 41 | 71.4 |
| Section III | 4 | 3 |  | 7 |  |  | 7 |  |  | 100 | 57 | 100 |
| Section IV | 1 | 5 | 1 | 7 |  |  | 7 |  |  | 100 | 57 | 100 |
| Section V | 7 |  |  | 6 | 1 |  | 5 | 2 |  | 100 | 49 | 85.7 |

**Appendix 3:** HEIA screening algorithm

| Bias towards HIA | To your knowledge: | Bias against HIA |
| --- | --- | --- |
| No | Are sufficient resources available to conduct the HIA? | Yes |
| No | Are the potential **-ve** health equity impacts associated with the activity likely to be serious? | Yes / don't know |
| No | Are the potential **-ve** health equity impacts likely to be serious if the activity doesn't proceed? | Yes / don't know |
| Yes | Is the activity of relatively short duration? | No |
| No / don't know | Is there scope to act on the recommendations of the HIA? | Yes |
| No | Are the potential **-ve** health equity impacts likely to be greater for vulnerable, marginalized, or disadvantaged groups? | Yes / don't know |
| No | Are there community concerns about potential health equity impacts? | Yes / don't know |
| No | Is there an evidence base to support the appraisal of the impacts? | Yes |
| No / don't know | Is the activity important in the context of corporate & environmental service priorities? | Yes |

**Appendix 4:** Details of the literature review conducted in the first stage of the study

- **The screening process of retrieved studies**

**Identification**

Studies obtained from PubMed/MedLine

N=265

Studies obtained from Scopus

N= 2003

Studies obtained from Google Scholar

N= 14633

The number of excluded studies by irrelevant topics and duplicate studies

N= 16658

Total retrieved studies

N= 16901

**Screening**

The number of excluded studies at the abstract review stage

N= 144

Reviewing the studies abstract

N=243

**Eligibility**

The number of excluded studies at the Full text review stage

N= 46

Full text studies to be critically evaluated

N= 99

**Included**

**Included studies**

**N=53**

- **Descriptive analysis of the included studies**

The descriptive analysis of the studies showed that in terms of the design, most of them had been conducted qualitatively (N=26). In addition, the studies conducted in this area had been mainly designed and implemented at the national level (N=31). During the past years (since 2016), more attention has been paid to performing health equity impact assessments.

| **Features** | | **Number**  **(N)** | **Proportion (%)** |
| --- | --- | --- | --- |
| **Study Design** | Qualitative | 26 | 49.5 |
|  | Quantitative | 7 | 13.2 |
|  | Mix methods | 14 | 26.4 |
|  | Review | 6 | 11.3 |
| **Study Level** | International | 15 | 28.3 |
|  | National | 31 | 58.5 |
|  | Local | 7 | 13.2 |
| **Year of Publication** | 2004-2009 | 5 | 9.4 |
|  | 2010-2015 | 18 | 33.9 |
|  | 2016-2021 | 30 | 57.6 |

- **Frequency of studies conducted to assess health equity impacts by different countries**
- **Brief data from analyzed articles**

| **Row** | **Title** | **Study design** | **Country** | **Study Level** | **Year of publication** | **Ref** |
| --- | --- | --- | --- | --- | --- | --- |
| 1 | Equity reporting: a framework for putting knowledge mobilization and health equity at the core of population health status reporting | Quantitative | Canada | National | 2018 | (68) |
| 2 | Evaluating the impact of equity-focused health impact assessment on health service planning: three case studies | Quantitative | Australia | National | 2014 | (67) |
| 3 | A rapid equity-focused health impact assessment of a policy implementation plan: An Australian case study and impact evaluation | Quantitative | Australia | National | 2011 | (66) |
| 4 | Capacity building process in environmental and health impact assessment for a Thai community | Mix method | Thailand | National | 2017 | (75) |
| 5 | Community Health Impact Assessment in Ghana: Contemporary Concepts and Practical Methods | Quantitative | Ghana | National | 2019 | (65) |
| 6 | Exploration of the functions of health impact assessment in real-world policymaking in the field of social health inequality: towards a conception of conceptual learning | Qualitative | Belgium | National | 2017 | (64) |
| 7 | Health Impact Assessment in the United States | Qualitative | USA | National | 2014 | (63) |
| 8 | A health impact assessment on the construction phase of a major hospital redevelopment | Mix method | Australia | Local | 2008 | (62) |
| 9 | Characteristics of health impact assessments reported in Australia and New Zealand 2005-2009 | Review | New Zealand | International | 2013 | (61) |
| 10 | Health equity impact assessment | Mix method | Australia | International | 2013 | (16) |
| 11 | Equity-focused health impact assessment of Portuguese tobacco control legislation | Mix method | Portugal | National | 2018 | (60) |
| 12 | Applying a health equity lens to evaluate and inform Policy | Review | USA | National | 2019 | (59) |
| 13 | Informing Investment to Reduce Inequalities: A Modelling Approach | Quantitative | UK | National | 2016 | (58) |
| 14 | A framework for regional primary health care to organise actions to address health inequities | Mix method | Australia | National | 2017 | (57) |
| 15 | Designing a toolkit for the assessment of Health in All Policies at a national scale in Iran | Qualitative | Iran | National | 2020 | (56) |
| 16 | Advancing Efforts to Achieve Health Equity: Equity Metrics for Health Impact Assessment Practice | Qualitative | USA | National | 2014 | (17) |
| 17 | Equity Impact Assessment of Interventions to Promote Physical Activity among Older Adults: A Logic Model Framework | Qualitative | Germany | National | 2019 | (55) |
| 18 | Framework for Participatory Quantitative Health Impact Assessment in Low- and Middle-Income Countries | Mix method | Spain | National | 2020 | (54) |
| 19 | Implementing Health Impact Assessment at National Level: An Experience in Iran | Qualitative | Iran | National | 2018 | (53) |
| 20 | Health impact assessment in planning: Development of the design for health HIA tools | Mix method | USA | National | 2010 | (52) |
| 21 | An equity tool for health impact assessments: Reflections from Mongolia | Qualitative | Mongolia | National | 2012 | (51) |
| 22 | Health impact assessment and urbanisation. Lessons from the NSW HIA Project | Review | Australia | National | 2007 | (50) |
| 23 | A systematic approach to equity assessment for digital health interventions: case example of mobile personal health records | Mix method | USA | National | 2019 | (49) |
| 24 | Integrating Health Equity Into Practice and Policy | Review | USA | National | 2016 | (48) |
| 25 | Critical considerations for the practical utility of health equity tools: a concept mapping study | Qualitative | Canada | International | 2018 | (47) |
| 26 | Development of a checklist to guide equity considerations in health technology assessment | Qualitative | Canada | International | 2021 | (46) |
| 27 | Health Equity Tools 2.0 2016 | Review | Canada | International | 2016 | (45) |
| 28 | Health Impact Assessment (HIA) for Planners: What Tools Are Useful? | Review | USA | International | 2010 | (44) |
| 29 | Development of Guidelines for Health Impact Assessment in Southern Italy | Qualitative | Italy | National | 2016 | (43) |
| 30 | Assessing Urban Health Inequities through a Multidimensional and Participatory Framework: Evidence from the EURO-HEALTHY Project | Qualitative | Portugal | local | 2020 | (42) |
| 31 | Developing a National Set of Health Equity Indicators Using a Consensus-Building Process | Mix method | Israel | National | 2020 | (41) |
| 32 | Development of an Urban Health Impact Assessment methodology: indicating the health equity impacts of urban policies | Mix method | UK | International | 2017 | (40) |
| 33 | The Development of a Rapid Health Impact Assessment Model for Songkhla Special Economic Zone (SEZ) Policy in Southern Thailand | Qualitative | Thailand | local | 2021 | (39) |
| 34 | Proposing a Framework for Health Impact Assessment in Iran | Mix method | Iran | National | 2015 | (38) |
| 35 | Using health impact assessment (HIA) to understand the wider health and wellbeing implications of policy decisions: the COVID-19 ‘staying at home and social distancing policy’ in Wales | Qualitative | UK | National | 2021 | (37) |
| 36 | Use of concurrent mixed methods combining concept mapping and focus groups to adapt a health equity tool in Canada | Qualitative | Canada | local | 2017 | (36) |
| 37 | The Role of Health Impact Assessment in promoting population health and health equity | Qualitative | Australia | National | 2009 | (35) |
| 38 | The gradient in health inequalities among families and children: A review of evaluation frameworks | Qualitative | UK | International | 2011 | (34) |
| 39 | The fit between health impact assessment and public policy: Practice meets theory | Qualitative | Australia | International | 2014 | (33) |
| 40 | The essential elements of health impact assessment and healthy public policy: a qualitative study of practitioner perspectives | Qualitative | Australia | National | 2012 | (32) |
| 41 | The Effectiveness of health impact assessment in influencing decision-making in Australia and New Zealand 2005–2009 | Qualitative | Australia | International | 2013 | (31) |
| 42 | Reducing health inequalities: the use of Health Impact Assessment on Rural Areas | Qualitative | Spain | National | 2015 | (30) |
| 43 | Using cost-effectiveness analysis to address health equity concerns | Quantitative | UK | National | 2017 | (29) |
| 44 | Process and impact evaluation of the Greater Christchurch Urban Development Strategy Health Impact Assessment | Qualitative | Australia | National | 2009 | (28) |
| 45 | GRADE equity guidelines 3: considering health equity in GRADE guideline development: rating the certainty of synthesized evidence | Mix method | Canada | International | 2017 | (27) |
| 46 | Embedding health equity strategically within built environments | Qualitative | Canada | local | 2018 | (26) |
| 47 | A Comparative Analysis of Health Impact Assessment Implementation Models in the Regions of Montérégie (Québec, Canada) and Nouvelle-Aquitaine (France) | Qualitative | France | International | 2020 | (25) |
| 48 | The application of urban health equity assessment and response tool (Urban HEART) in Tehran; concepts and framework | Qualitative | Iran | local | 2010 | (24) |
| 49 | The impact and effectiveness of health impact assessment: A conceptual framework | Qualitative | Australia | local | 2013 | (23) |
| 50 | What makes health impact assessments successful? Factors contributing to effectiveness in Australia and New Zealand | Mix method | Australia | International | 2015 | (22) |
| 51 | GRADE Equity Guidelines 4: Guidance on how to assess and address health equity | Mix method | Canada | International | 2017 | (76) |
| 52 | HEIA tools: inclusion of migrants in health policy in Canada | Qualitative | Canada | National | 2018 | (77) |
| 53 | Quantitative health impact assessment: current practice and future directions | Quantitative | Netherlands | International | 2004 | (21) |

**Appendix 5:** Results of the pilot study; HEIA report for “the policy of removing the subsidies from some basic food products”

| **Section I: Policy Introduction** | | | | | | | | |
| --- | --- | --- | --- | --- | --- | --- | --- | --- |
| **Title:** The policy of removing the subsidies from some basic food products | | | | | | | | |
| **Main objective(s):**   - To compensate for the negative effects of the increase in the global price of imported livestock and agricultural inputs and occurrence of drought in the current year, and to ensure an adequate supply of basic goods, permanent inputs, medicine, and medical consumables - Elimination of preferential currency allocated to food - Targeted provision of food support packages | | | | | | | | |
| **Policy type:**  Plan  Bill  Circular  General policies Others; Name: ................................ | | | | | | | | |
| **Policy level:**  National  Provincial Local | | | | | | | | |
| **Department(s) / institution(s) proposing the policy**: Presidential office | | | | | | | | |
| **Department(s) affected by the policy:**  Education Communication and Information Technology Economic and Property  Health (Healthcare) Social welfare Agriculture  Justice Road and urban development Industry, mining, and trade  Science, research and technology  Sports Private sector: All importing companies and factories producing food☒  Others (name):.............. | | | | | | | | |
| **Trustee of policy implementation:** Government | | | | | | | | |
| **Date of policy proposal in parliament:** 11/19/2021 | | | | | | | | |
| **Section II: Managing the HEIA of Policy** | | | | | | | | |
| **Evaluation type:**  Desk-based ☐ Rapid ☒ Middle ☐ Comprehensive☐ | | | | | | | | |
| **Credit for assessment (Rials):** $0 | | | | | | | | |
| **Research Team & authors:**  Natural person: ................  ☒ Juridical entity: Health Equity Research Center (HERC), Tehran University of Medical Sciences (TUMS) | | | | | | | | |
| **Number of team members**: 6 | | | | | | | | |
| **Time duration:** 5 weeks | | | | | | | | |
| **Type of reporting and dissemination**  Formal report  Executive summary  Community report  Briefing report  Others: Checklist of assessment report on the impact of policies on health equity☒ | | | | | | | | |
| Is there a checklist attachment?  Yes☐  No☐  If "yes", the number of attachments: 1  Attachment Title: full report of HEIA of the policy of Removing the subsidies from some basic food products | | | | | | | | |
| **Section III: Scope of the affected population** | | | | | | | | |
|  | | | | **Population influenced by positive impacts** | | | | **Population influenced by negative impacts** |
| **Introduction of affected population:** | | | | Domestic food producers (whose productions have no dependence on imported products) | | | | Total population of the country |
| **The number of affected people:** | | | | 2,540 food industrial workshops  216000 people employed in these industries  (The numbers are approximate) | | | | 87.92 million people (based on 2021 statistics) |
| **Distribution of affected population:** | | | | | | | | |
| **Age** | | Under 5 years old | |  | | | |  |
|  |  | 5-14 years old | |  | | | |  |
|  |  | 15-49 years old | |  | | | |  |
|  |  | 50-69 years old | |  | | | |  |
|  |  | Over 70 years old | |  | | | |  |
| **Gender** | | Male | |  | | | |  |
|  |  | Female | |  | | | |  |
| **Formal education level** | | Illiterate | |  | | | |  |
|  |  | Elementary | |  | | | |  |
|  |  | High school | |  | | | |  |
|  |  | Bachelor and senior | |  | | | |  |
|  |  | Ph.D. and above | |  | | | |  |
| **Economic quintiles** | | First quintile | |  | | | |  |
|  |  | Second quintile | |  | | | |  |
|  |  | Third quintile | |  | | | |  |
|  |  | Forth quintile | |  | | | |  |
|  |  | Fifth quintile | |  | | | |  |
| **Vulnerable groups** | | Children <19 years | |  | | | |  |
|  |  | Elderly > 60 years | |  | | | |  |
|  |  | Handicapped, physically/mentally disabled/special patients | |  | | | |  |
|  |  | Female-headed households | |  | | | |  |
| **Geographical region** | | Urban | |  | | | |  |
|  |  | Rural | |  | | | |  |
|  |  | Marginalization | |  | | | |  |
|  |  | Informal settlements | |  | | | |  |
| **Row** | **Section IV: HEIA results** | | | | | | | |
|  | **Health equity impact** | | **Direction** | **Affected population** | **Impact duration** | | **Impact likelihood** | **Impact severity/magnitude** |
| 1 | Malnutrition prevalence rate | | Increase | All people, especially:   - Vulnerable groups - First, second, and third economic quintiles - Rural areas and disadvantaged provinces | Medium term | | Causal relationship established | Very high |
| 2 | Prevalence of underweight | | Increase | - Children under 5 years old | Long term | | Causal relationship established | High |
| 3 | Prevalence of short stature | | Increase | children under 5 years old | Long term | | Causal relationship established | High |
| **General assessment of policy impact on health equity:**  Low  Medium High Very high | | | | | | | | |
| **Section V: Recommendations** | | | | | | | | |
| **Strategies to reduce negative impacts and increase positive ones** | | | | | | **Advantages** | | **Disadvantages / Limitations** |
| Establishing strict regulatory institutions and hiring technical experts to control fluctuations and growth of product prices in the final market | | | | | | Increasing accountability  Increasing transparency | | Obligation to spend money for creating regulatory, informational, and administrative infrastructures |
| Establishing strict monitoring institutions to clarify the allocation of preferential currencies to the amount and types of food imports (in case of continued currency allocation) | | | | | | Increasing accountability  Increasing transparency | | Obligation to spend money for creating regulatory, informational, and administrative infrastructures |
| Taking advantage of alternative policies, including targeted and temporary cash-goods payment (eligible groups), | | | | | | Ensuring that the disadvantaged are less harmed | | Increasing liquidity in the market |
| Proper regulation of the food market and stability in the currency market | | | | | | Controlling the supply and demand in food market and making people less affected by the increase in food inflation | | - |
| The intervention of the government in the strategic food market and its proper supervision for systematic distribution of food | | | | | | Equity in access to food by different groups in different regions | | Disruption of market rules |
| Applying appropriate policies to increase income; This policy can cause consumers to diversify their food baskets and shift from consuming grains and low-nutritional value products to high-nutritional value foods such as meat, dairy products, fruits, and vegetables. | | | | | | Increasing people’ purchasing power and thus, decreasing their vulnerability to inflation fluctuations | | This is a very interdisciplinary policy, and achieving it is necessary, but it cannot be considered a quick solution to solve the problem. |
| **Targeted subsidies to improve food security can be provided in the following two ways:**  - Commodity basket: Food support policy is suggested using the commodity basket for low-income groups. To implement this policy, it is necessary to first identify the target community and then estimate its implementation cost based on the number of people involved in the plan and the type of items considered to be distributed.  - Coupons and electronic invoicing: An electronic invoice is a commercial document issued by the seller of goods or services to the buyer and includes information such as agreed price, amount of goods or services, taxes, etc. The service provider is required to comply with all standards announced by the government when issuing this invoice. He must then confirm the form by completing it through electronic signature and sending it to the service recipient. After the electronic signature by the service provider, it will not be possible to edit the invoice. All these steps will be carried out in a network under the supervision of the tax authority and the information will be sent to the tax office instantly. | | | | | | Use of support baskets in a targeted and comprehensive manner leads to increased food security, especially for deprived classes, provided that the requirements, including the adequacy of support packages and universality for all eligible people, are taken into account.  In the implementation of this policy, using coupons and electronic invoicing is more transparent and efficient. | | Due to economic rents created during the implementation of this policy, the waste of resources will increase. This means that financial corruption may occur in using the allocated budget in the absence of powerful supervision during the preparation and distribution of support packages.  The use of coupons and electronic invoicing requires sub-costs as well as powerful information and monitoring infrastructures. |
